# Green Recycled Aggregate in Concrete: Feasibility Study

**DOI:** 10.3390/ma18030488

**Published:** 2025-01-22

**Authors:** Magdalena Bardan, Lech Czarnecki

**Affiliations:** 1 Construction Materials Engineering Department, Building Research Institute, 00-611 Warsaw, Poland; m.bardan@itb.pl; 2 Scientific Secretary, Building Research Institute, 00-611 Warsaw, Poland

**Keywords:** recycled aggregate concrete, green materials, sustainable construction industry, circular economy, AI-assisted analysis

## Abstract

With increasing concrete production, CO_2_ emissions rise, and natural resources deplete, creating a need for new material solutions. This article analyzes the feasibility of using green materials, like recycled aggregate (RA) from construction and demolition waste (CDW) to be incorporated into concrete (RAC). The objective of this paper is to determine that the use of RA ensures receiving sustainable concrete in comparison with NA and LA. The sustainability assessment was conducted based on an analysis of the life cycle in terms of the environmental, economic, and public perception aspects. Additionally, the analysis was extended to include two newly introduced indicators: quality of aggregates and concrete performance. A proprietary scoring method based on ideal aggregate characteristics was used, which was enhanced by innovative multidimensional analysis, with credits assigned based on a literature review conducted using artificial intelligence (AI) statistical tools to partially assist in the selection of items. The results could even show that RA outperformed natural aggregates (NA) and artificial (light) aggregates (LA) in the environmental (over 80% of the results) results as well as the economic (over 65%) and public perception categories (over 80%). However, RA ranked second behind NA in terms of quality aggregates and concrete performance, with LA scoring lowest. The results highlight RAC as a satisfactory sustainable option compared with NAC, supporting the circular economy by reducing waste, emissions, and resource consumption. The best solution would be hybrid concrete containing a partial substitute for natural aggregates in the form of recycled aggregates, enabling the advantages of both types of aggregates to complement each other and offset their limitations.

## 1. Introduction

Continuous population growth exerts considerable pressure on the development and expansion of the construction sector [1]. As a result, the global demand for aggregates is constantly growing, and it is estimated that by 2030, it will reach 50–55 billion tons [2]. Concrete is the most manufactured product worldwide and has dominated the market since the beginning of the 20th century. Its production is predicted to continue to grow in response to the continuous demand in the construction sector [3,4,5]. Consequently, the use of natural resources, such as aggregates [6], which constitute about 70% of the volume of concrete mixes, will continue to be intensive [4,7]. These factors are in line with the view described previously and bring us closer to the thermodynamic barrier, which is the point beyond which natural resources and ecological systems are not able to regenerate themselves effectively [8,9]. If our actions are bringing us closer to this barrier, then sustainable development becomes not only an ethical or economic choice but also a necessity dictated by the laws of thermodynamics. This means that we must act in a way which minimizes the increase in entropy (i.e., in a way which strives for balance and minimization of the negative environmental impact). Therefore, actions are being implemented to protect the environment, promote sustainable resource use, and manage waste in line with the circular economy (CE) model [10].

Recycled aggregates (RA), resulting from the crushing of rubble derived from the demolition of buildings, including those destroyed as a result of natural disasters, such as floods or windstorms, show significant potential as a substitute for natural aggregates (NA) in the production of concrete [11,12,13]. RA are actually NA surrounded by the original cement mortar [12]. Their potential is particularly important in the context of reducing the exploitation of natural resources and minimizing the amount of waste sent to landfills (CDW) [3,14,15,16]. Despite numerous studies demonstrating the benefits of using recycled aggregates (RA), their practical application in structural concrete on an industrial scale remains limited [17]. In this context, one question arises: “Does recycled concrete (recycled aggregates) align with the circular economy model?”. Artificial intelligence responds “yes” with 100% certainty [18]. Critical thinking, however, suggests that there are some factors inhibiting this process, necessitating further analysis of various aspects which point to new directions in the development of sustainable concrete with recycled aggregates [3].

This article is a review paper which focuses on the feasibility study of recycled aggregate concrete (RAC), analyzing its potential as an alternative to natural aggregate concrete (NAC). This study identifies the key features of an ideal product supporting sustainable development, enabling evaluation of the opportunities, barriers, and challenges related to RAC implementation. This work expands the existing knowledge by extending analyses to include the properties of RA, RAC, and their life cycles, with an emphasis on identifying the reasons for limited practical applications. The authors’ multicriteria analysis was based on the original data extraction, and the interpretation conducted on this topic relied on innovative data extraction and interpretation, allowing for the identification of potential obstacles and opportunities in applying these materials in sustainable construction.

The evaluation took into account the following five aspects: environmental, economy, public perception, as well as quality aggregates and concrete performance. The analysis compared RA and NA in the context of their use in the production of concrete, which was a key object of this study. In addition, the analysis was extended to LA and LAC. In the analysis, priority was given to NA and RA. Such a distribution resulted mainly from the need to emphasize the importance of alternatives to NA and RA while taking into account the limitations to the use of LA in structural and non-structural concretes compared with NA and RA [19]. In order to assess these factors, an original scoring method was adopted, based on the scale of “ideal characteristics of aggregates for concrete” in a given aspect, in order to illustrate the results and facilitate their interpretation. In summary, the objective of this paper is to demonstrate that the use of RA ensures the production of sustainable concrete compared with NA and LA.

## 2. Materials and Methods

### 2.1. Methods

The feasibility study described in this article outlines a process aimed at achieving a goal, resulting in a multicriteria analysis which allows for a comprehensive assessment of the synergy between key aspects defining the sustainability of products, with potential for practical application in the concrete industry. For clarity, the process was divided into four stages, with each being built directly upon the previous one.

**Figure 1:**
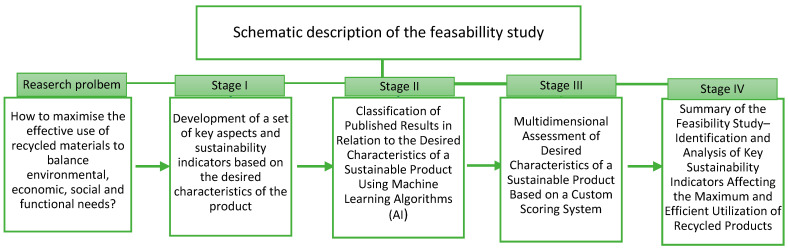
Schematic representation of the feasibility study showcasing the cascading structure of the stages, leading to their integration within the framework of multicriteria analysis and enabling the identification of key sustainability indicators.

Stage I involved identifying the key aspects of product sustainability and the indicators describing them. Stage II focused on data collection and implementation using modern tools, such as artificial intelligence algorithms. Based on this, in Stage III, evaluation criteria were established, and the collected data were analyzed, leading to the development of a comprehensive multicriteria assessment model. In Stage IV, a multicriteria analysis was conducted, identifying and visualizing the key sustainability indicators which impact the maximum and effective utilization of recycled products. Each stage was carried out in a cascading manner, as illustrated in Figure 1.

To ensure structural consistency and a logical presentation of the study results, the individual stages of the schematic are detailed in the Section 2 and subsequently discussed in the same order in the Section 3.

Table 1 was included in the article to provide readers with a clear and consistent understanding of the abbreviations and terms related to the subject matter.

**Stage I: Development of a set of key aspects and sustainability indicators based on the desired characteristics of the product**

The idea of sustainable development assumes that the needs of contemporary society will be met without limiting the ability of future generations to fulfil their own needs. The methodology for selecting aspects and their corresponding indicators was based on a systematic review of the scientific literature conducted by the authors. It was noted that the key features describing sustainability included environmental, economic, and social aspects [9,10,12,14]. Significant emphasis was placed on the assessment of aggregate quality through proposals defining evaluation criteria and their impact on the properties of concrete [11,20,21,22,23,24]. Therefore, the decision was made to select five aspects.

For every aspect, three of the most frequently recurring indicators were selected, enabling identification of the advantages and disadvantages of the material solutions used, which were typically described in publications through life cycle analysis. The authors’ concept involved extending these analyses to include characteristics describing the quality of the aggregates and the concretes containing them, reflecting the significant interest and attention devoted to these issues by the authors. Additionally, the focus was placed on identifying the three most important indicators for each aspect to enable a comprehensive evaluation of material solutions. In the context of environmental aspects, special attention was paid to the impact of concrete on CO_2_ emissions from production, the extraction of natural resources, and the storage of waste in landfills. The lower the emissions and consumption of raw materials, the more advantageous the features of the product are from the perspective of sustainability. In terms of economic factors, the costs of production, transport, and storage of waste were analyzed, with lower costs meaning that the product is more competitive and attractive. In turn, in the context of public perception, the higher the level of social acceptance, regulatory support, and the possibility of implementing innovative solutions, the better its assessment. Significant attention was also paid to analyzing the impact of the quality of aggregates on the strength and durability of concretes where they were added. Special attention was paid to the grain structure in the context of its ability to absorb water, its density, as well as the type of pollution accompanying the production process. Possible application of aggregates in concretes of various classes was also analyzed. The last aspect considered was the impact of the types of aggregates on the rheology of a concrete mix and the properties of hardened concrete, which included a comparison of design assumptions.

To summarize this stage, a new approach was applied to systematize individual indicators in every aspect of sustainability, being developed in such a way as to indicate the desired features against the characteristics of a sustainable product (i.e., whether it reduced CO_2_ emissions, energy costs, and the use of natural resources). A summary of the desired features of a sustainable construction product is listed in Table 2.

**Stage II: Classification of the results in relation to the desired characteristics of a sustainable product using machine learning (AI) algorithms**

To streamline the process of retrieving research from extensive databases, a machine learning tool (AI), specifically consensus.app [18], was utilized. AI was employed solely for the random selection of works, and its role ended at this stage without influencing subsequent analyses or evaluations. The tool scanned over 500 databases (Semantic Scholar, a system collaborating with academic journals) and filtered the results, providing access to publications relevant to the research question and enabling an unbiased selection of sources. The algorithms combine traditional search methods with advanced language models (LLMs) and vector search technology. Both automated algorithms and traditional literature selection methods carry the risk of incomplete results, highlighting the need for diligence and a critical approach when evaluating available sources. Therefore, the content of each article was reviewed for alignment with the research question, and conclusions were analyzed to provide insights into the impact of RA, NA, and LA on the respective indicator.

One question was posed for each aspect of the aggregates (RA, NA, and LA). In response to the posed questions, the AI provided a collection of articles. A total of 150 answers were obtained. In the first query, the search engine displayed 10 articles, and the review was limited to this number. Each article was assigned a DOI number, and the number of citations was recorded.

In some cases, articles overlapped with respect to a given aspect or aggregate, indicating relevance to several indicators simultaneously. For RA and NA, the content of these publications allowed for the identification of critical information. However, for LA, due to the smaller literature base, duplicates occurred more frequently, and their content did not enable evaluation across all specified indicators. One example is the lack of data regarding societal impact in the context of transparency in documentation. This situation demonstrates that literature search methods using AI tools are most effective when applied to extensive bibliographic resources.

The purpose of the classification of the published results was to assess how close each type of aggregates and concrete with their addition was to a sustainable product with the desired characteristics, depending on the aspect analyzed. It was assumed that the ideal sustainable product would score the maximum number of points in a given aspect (i.e., 10). To assess this, open-ended analytical questions were formulated. One question was asked for each aspect, with the questions having identical content and differing in the subject of the analysis in relation to the three types of aggregates.

A total of 15 questions were asked (Σ_Q_ = A × XA, where Σ_Q_ is the total number of questions; A is the number of aspects (5), specifically environmental, economic, public perception, quality of aggregates, and concrete performance; and XAC is the number of types of aggregates (3), namely recycled aggregate concrete (RAC); natural aggregate concrete (NAC), and artificial (or light) aggregate concrete (LAC)). In the case of recycled aggregates, this was clarified to be crushed concrete, since many different materials derived from recycling exist.

The set of questions asked is given below.

Aspect No. 1 (Environmental): How do recycled (crushed concrete), natural, and artificial aggregates impact the natural environment?

Aspect No. 2 (Economic): How do recycled (crushed concrete), natural, and artificial aggregates impact the economics of concrete production?

Aspect No. 3 (Public Perception): How do recycled (crushed concrete), natural, and artificial aggregates impact the economics of public perception?

Aspect No. 4 (Quality Aggregate): What impacts do the parameters of recycled, natural, and artificial aggregates have on their quality?

Aspect No. 5 (Concrete Performance): What is the impact of recycled, natural, and artificial aggregates on the performance of concrete?

To answer the posed questions, a literature review was conducted using an AI tool which selected publications from a wide database of sources related to RA and concretes containing them. The subject of the study was the data obtained from this review. A total of 150 responses were obtained (Σ_A_= Σ_Q_ × 10, where Σ_A_ is the total number of answers and 10 is a fixed number of articles received for a given question).

The articles were grouped according to the answers to the questions, specifically “yes, it has a positive impact”, “it is possible that it has a positive impact”, and “no, it has a negative impact” for a given indicator. The uncertainty of the classification method was related to a subjective assessment. To reduce this as much as possible, criteria for data extraction were developed, which are described in Table 2. The articles were assigned to the “yes, it has a positive impact” category if the author, on the basis of their own laboratory tests or literature review, conclusively proved a positive impact on a given indicator in a particular aspect. The “it is possible that it has a positive impact” category was applied when the studies indicated a potentially positive impact, but there were no definitive conclusions. In turn, “no, it has a negative impact” meant that the author, on the basis of laboratory tests or a literature review, demonstrated a negative impact on a given indicator in a particular aspect. In some cases, the articles overlapped, pointing to a positive impact on several aspects. The assessment of the indicators for particular aspects was auxiliary in nature, and ultimately, the points were counted in the context of the entire aspect.

The points assigned to each aspect were counted, and their percentage share was calculated. In consecutive stages, all assessments were analyzed in order to arrive at a complete picture of the impact of individual aspects on a given indicator. Positive (green color), potentially positive (orange color), and negative (red color) results were taken into account, which allowed for a comprehensive assessment of the potential benefits and risks. The final stage of the analysis focused on the positive impact, comparing the results with the criteria determining the ideal construction product in order to assess its compliance with the requirements of sustainable development.

**Stage III: Multidimensional assessment of the desirable characteristics of a sustainable product based on the authors’ original scoring system**

In the third stage, RA, NA, and LA and the concretes they were added to were assessed using a scoring method which referred to the desired characteristics of sustainable concrete. An original method of awarding points was developed, called ideal characteristics of aggregates for concrete, for each given aspect: environmental, economy, public perception, quality aggregates, and concrete performance. Based on the percentage share of “yes”, “no”, and “possible” answers given by scientists regarding various aspects of sustainability, which in the third stage were summarized using artificial intelligence (AI) tools, ranges were created, and points were assigned to appropriate ranges. The conversion of percentage points into score points facilitated a comparison of the different datasets contained in Table 3. The percentage range of results was divided and defined as follows: 80–100% = 5 points, 60–79% = 4 points, 40–59% = 3 points, 20–39% = 2 points, and 0–19% = 1 point. The assessment consisted of awarding points on a scale from 1 to 5 in the context of sustainability in a given aspect to various types of aggregates and concretes where they were added, where higher values denote more favorable characteristics. This scale is as follows: 5 means highly beneficial, 4 is beneficial, 3 is acceptable, 2 is permissible, and 1 is unacceptable. The ideal sustainable product scored the highest in a given aspect.

**Stage IV: Identification and analysis of the key sustainability indicators affecting the maximum and effective use of recycled products**

The multifaceted analysis carried out at this stage made it possible to identify critical indicators, which led to more detailed analysis and proposals for optimization. Thanks to the distribution of points in individual aspects and their graphic visualization, it was possible to analyze the disadvantages and advantages of a given solution. Such an analysis allowed for quick identification of areas for improvement and distinguished the most favorable characteristics of materials. As a result, it was possible to make informed decisions which support the optimization of processes and enhance the efficient use of construction materials.

### 2.2. Materials

**Figure 2:**
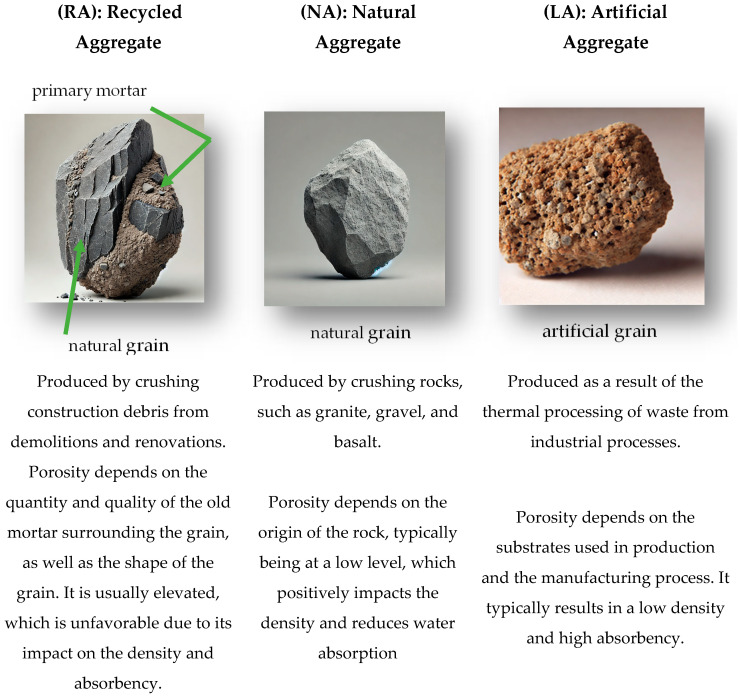
Coarse aggregate grain structure.

The subject of the feasibility study encompassed three types of aggregates—RA, NA, and LA—as well as the concretes containing them. Specifically, it focused on data obtained from a literature review on these materials. An overview of the aggregate characteristics, which according to the requirements of the standard [25,26,27] can be used in concrete production, is presented in Figure 2. The images presented in the figure are illustrative and do not represent actual depictions. Their purpose is to provide a visual comparison of the aggregates and highlight key differences in their structure which directly influence the properties of the concrete containing them. The approximate dimension is 16 mm.

In 2022, approximately 2500 million tons of fine and coarse aggregates were extracted in Europe alone. By comparison, about 295 million tons of recycled aggregates (RA) were produced. Germany (80 million tons), France (65.9), and the Netherlands (23.5) emerged as clear leaders in the production of these aggregates. Lightweight aggregates (LA) ranked third, with a production volume of 55 million tons [28].

Natural aggregates (NA), according to the definition of sustainable construction, are resources which should be preserved, as demand for their use in the construction sector continues to rise. NA are produced through the mechanical processing of rocks of various origins. RA, on the other hand, are products derived from the processing of materials previously used in construction. As a result, RA have a significantly more porous structure compared with NA, as the grains are encased in old cement mortar. RA are characterized by a heterogeneous structure and variable physicochemical properties. Each batch of material obtained from building demolition provides a unique combination of components, adding further variability. For this reason, RA require detailed supervision and more frequent quality control to ensure consistency in technical parameters. The standard [25] indicates the possibilities of using coarse recycled aggregates in concrete production, dividing them into two types and specifying the mass percentages of their use depending on the exposure classes (type A or B).

LA are low-density products made from mineral raw materials such as clay, shale, marl, fly ash, or blast furnace slag. These raw materials are subjected to thermal and mechanical processing, resulting in a porous structure (low density). Due to these processes, LA are characterized by their low mass, making them particularly useful in the production of lightweight materials, such as lightweight concrete.

There are uniform formal conditions which allow the introduction of all three types of aggregates into circulation within the European Union and in countries that implement international standards and regulations related to the circular economy, such as EU CPR 305/2011. The harmonized standards (European Committee for Standardization, 2008) and (European Committee for Standardization CEN, 2016) are assigned to NA and RA, respectively, as well as LA. To better understand the differences in regulatory transparency, it is necessary to focus on two key stages.

The first stage concerns the loss of waste status, covering the production of RA and LA directly before the type testing described in the harmonized standards. This process does not apply to natural aggregates. RA are produced as crushed concrete resulting from construction demolitions, such as concrete, bricks, and asphalt. The second stage involves testing and certification. The harmonized standards are classification-oriented; they define classes and material categories which aggregates can meet but do not include guidelines for specific applications. These standards classify aggregates based on their origin (NA, RA, or LA), size (grain size defining the fraction), type (fine, coarse, continuously graded, or filler), and density (lightweight or heavyweight). They also specify testing methods which enable the precise definition of aggregate characteristics.

The technical guidelines for specific applications can be found in the standard [25], which contains different requirements for the three types of aggregates. NA and RA can mostly be tested using the same methods and meet identical requirements in geometric categories, such as the shape index (SI ≤ 55) or flakiness (FI ≤ 50), and in physical categories, such as resistance to fragmentation (LA ≤ 50) or thermal shock (SZ ≤ 32). For LA, however, the testing methodology needs to be modified. Additionally, all requirements, except for the contents of acid-soluble sulfates and sulfur, are declared by the manufacturer. It is worth noting that the categories described above characterize low-quality aggregates, meaning practically all types of aggregates can fit within them. Developing more detailed quality criteria would be extremely beneficial to better define their properties and potential applications.

## 3. Results

### 3.1. Classification of the Published Results in Relation to the Desired Characteristics of the Sustainable Product Using Machine Learning (AI) Algorithms

Based on the formulated research questions concerning the environmental, economic, public perception, as well as quality of aggregates and concrete performance aspects, 10 publications were analyzed for each type of aggregate—RA, NA, and LA—in relation to each of the specified aspects. Within the content of the articles, three indicators were identified for each aspect. Based on the collected data, the impact was classified into three evaluation categories: positive impact, potentially positive impact, and negative impact. The obtained scores were aggregated and subjected to statistical analysis, allowing the determination of the percentage share of each impact category. Table 4 presents a detailed distribution of the results for the three types of aggregates, reflecting their impact on specific aspects of sustainable development. The maximum number of points for each aspect was 10, which corresponded to 100%.

#### 3.1.1. Aspect No. 1: Environmental

**Figure 3:**
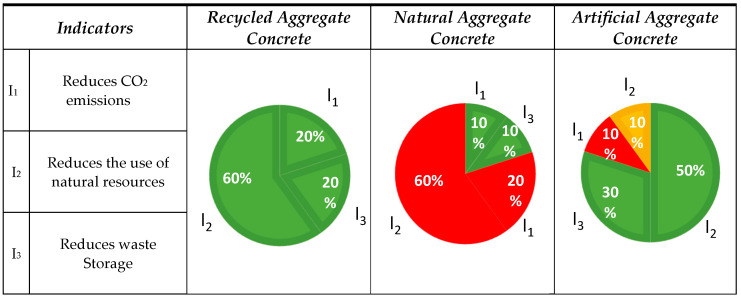
The classification of the published results is presented for the environmental aspect for the aggregates (RA, NA, and LA) and the concretes containing them. Publications assigned to indicators, RA: I_1_ [29,30]; I_2_ [17,31,32,33,34,35]; I_3_ [4,36], NA: I_1_ [35,37,38]; I_2_ [31,39,40,41,42,43]; I_2_ [31,39,40,41,42,43], LA: I_1_ [44]; I_2_ [7,19,45,46,47,48]; I_3_ [11,49,50].

The environmental impact was assessed by identifying three interrelated indicators: reduction in CO_2_ emissions (I_1_), protection of natural aggregates resources (I_2_), and reduction in the amount of waste stored in landfills (I_3_). The classification of the published results is presented in Figure 3.

The analysis demonstrated that in the case of RA, all 10 of the publications studied (10/10) clearly indicated its positive impact in the context of environmental protection, taking into account the three key indicators (I_1_, I_2_, and I_3_). This result represents the highest possible rating, with 100% of the responses evaluated as having a positive impact. In the case of NA, out of all of the publications, two (2/10) indicated its positive environmental impact. A complementary analysis of LA showed more positive opinions than in the case of NA (+6) but fewer than RA (−2). Eight of the 10 publications studied (8/10) indicated their positive impact.

Indicator I_1_: Two articles [29,30] on RA presented a positive attitude toward the reduction in carbon dioxide emissions, emphasizing the importance of factors such as local transport of aggregates (close distances of 15–25 km) and reiterating the importance of the effective amount of RA in the concrete mix and the optimization of its composition. Carbon dioxide emissions may increase when natural coarse aggregates are replaced by RA in proportions of 50% and 100%. This is related to the addition of cement due to a decrease in strength above 45 MPa and thus the addition of an increased amount of cement, which affects CO_2_ emissions [29]. Opinions on carbon dioxide emissions for NA were divided; one publication indicated a positive impact [35], while two [37,38] presented a negative effect. The latter concerns the production of Nas, which generates significant CO_2_ emissions, amounting to 4.45 kg CO_2_ eq/ton, and consumes 2.30 kgoe/ton of primary energy [38]. One positive opinion for NA concerns CO_2_ reduction through the use of a solution combining NA and RA, as well as minimizing transportation distances [35]. One publication on LA demonstrated a negative impact in the context of carbon dioxide emissions. The level of emissions related to the production of aggregates depends on what method of production is used [44].

Indicator I_2_: Six publications on RA confirmed the beneficial effect of reducing the consumption of natural resources [17,31,32,33,34,35]. However, it was emphasized that the quantity share of RA would allow for assessing the effectiveness of this solution. Coarse recycled aggregates have the lowest environmental impact, while NA (crushed and sourced directly from natural raw materials) have the greatest impact [31]. A total of eight negative opinions on NA were recorded, six of which concerned their destructive impact on ecosystems [37,39,40]. The extraction and use of NA significantly harm the environment, especially in the context of land degradation [37]. This process leads to the formation of large excavations which transform the natural landscape, destroying natural habitats and disrupting the functioning of local ecosystems and biodiversity [42,43]. Five publications on LA highlighted the potential to reduce natural resource extraction by replacing NA with industrial waste, presenting a viable alternative, although the application of LA is mainly limited to lightweight concrete [11,49]. One publication indicated a potentially positive environmental impact from the analyzed solution, emphasizing that its effectiveness depends on the properties of the LA [47].

Indicator I_3_: Two articles [33,36] on RA indicated a reduction in the amount of waste stored in landfills. CDW exceeds 10 billion tons per year, and it has great potential as an RA in the production of concrete due to the high raw material value of its components [36]. It is worth noting the carbon footprint of landfills because during the decomposition of organic waste, they emit a greenhouse gas: methane. Additional emissions are also generated by the transport processes and waste management [33]. One positive opinion for NA highlighted their favorable mechanical properties, which enable the use of RA in concrete mixes [51]. Three publications highlighted the potential of LA in recycling industrial and agricultural waste, such as nut shells, ceramic waste, concrete, and glass, as alternative aggregates, enabling landfill reduction [11,49,50].

#### 3.1.2. Aspect No. 2: Economic

**Figure 4:**
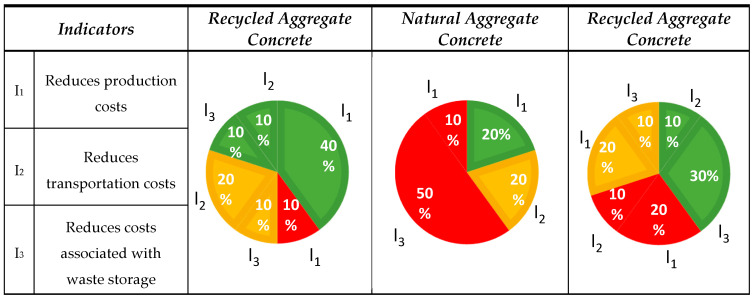
The classification of the published results is presented for the economic aspect for aggregates (RA, NA, and LA) and the concretes containing them. Publications assigned to indicators, RA: I_1_ [34,52,53,54,55]; I_2_ [31,32,37]; I_3_ [17,56], NA: I_1_ [32,56,57]; I_2_ [31,37]; I_3_ [34,58,59,60,61], LA: I_1_ [44,46,62,63]; I_2_ [19,64]; I_3_ [7,65,66,67].

The environmental impact was assessed using three interdependent indicators: the costs of production (I_1_), transport (I_2_), and waste storage (I_3_). The classification of the published results is presented in Figure 4.

The distribution of results regarding the impact of RA on concrete production costs was not as clear cut as its environmental impact, where all 10 analyzed publications indicated a positive effect. For the economic aspect, the result was 40% lower. The positive impact of RA on the reduction in concrete production costs was confirmed in 6 out of the 10 works analyzed (6/10) [17,34,52,53,54,55]. The reduction in production costs was the most discussed aspect in the analyzed publications. The analysis of publications examining the impact of NA on the costs of concrete production indicated various positions; out of all of the assessments recorded, six were negative [32,34,58,59,60,61], two positive [56,57], and two indicated a potential positive impact [31,37]. Only 2 out of 10 (2/10) publications indicated that NA may help reduce costs, with negative opinions prevailing. This was the opposite of RA, where 60% of the opinions were positive. The analysis of publications on the impact of LA on concrete production costs also indicated diverse opinions, as with NA. A total of 4 out of 10 (4/10) publications indicated this, which was 20% more than in the case of NA but, at the same time, 40% less than in the case of RA.

Indicator I_1_: The potential for cost reduction for RA was indicated in four works [34,53,54,55], which focused on the production processes and the degree to which RA substituted NA. One publication [52] demonstrated a negative impact when large proportions of RA substituted NA [54]. The costs include the purchase and operation of concrete crushing equipment, as well as increased consumption of cement and additives, which at higher replacement levels lead to an increase in concrete production costs [34,53]. An appropriate replacement level (30–100%) can reduce the production costs of recycled aggregate concrete (RAC) by 9–28% compared with NAC [52,55]. One publication highlighted the high cost of concretes with NA, especially when locally available RA reduce transport and processing costs [46]. Two publications suggested that the addition of RA can mitigate this effect [31,37]. Two publications for LA expressed negative opinions about their production methods; the first highlighted their limited production capacity [46], and the second pointed to high energy demands, depending on the manufacturing technologies used [66]. Two publications indicated a potential positive impact, as the appropriate type of waste can be transformed into useful materials [19,64].

Indicator I_2_: The second factor to consider was the transport of RA, and three opinions were offered: one indicating a positive impact [37] and two suggesting a potential positive impact [31,32]. The economic efficiency of using RCA largely depends on the proximity of the source of recycling to the place of application, which may cut the costs of transport [31,37]. RCA, if sourced within a short distance from a demolition site, costs approximately 74% of the NA price, which indicates its competitiveness [32]. Two publications on NA indicated a potential cost reduction in the case of locally available deposits. One publication on LA highlighted a positive impact on reducing transportation costs due to its lower mass (low density). However, the lack of production facilities nearby may increase transportation costs, as noted in one publication [19].

Indicator I_3_: Savings connected with disposal costs were emphasized by two publications; one indicated a positive impact from recycling [56], eliminating the necessity for waste storage, and the other suggested a potentially possible reduction of these costs, provided that specific technological and organizational requirements are met [17]. Using 20% recycled concrete aggregates instead of NA in concrete mixes has no significant impact on the production costs or the environment. In addition, the use of RA has certain benefits, such as reducing the amount of waste by 8% and reducing the consumption of non-renewable natural (abiotic) resources by 10.6% [56]. The issues analyzed for NA focused primarily on the intensive extraction of NA at the expense of recycling RA, which in many cases end up in landfills [34,58,59,60,61]. Three positive opinions for LA related to the reduction in costs associated with waste storage and recycling [7,65,66]. One publication highlighted a potential positive impact on reducing landfill waste. Industrial waste, such as dust, fly ash, and slag, often ends up in landfills, generating high storage costs [48].

#### 3.1.3. Aspect No. 3: Public Perception

**Figure 5:**
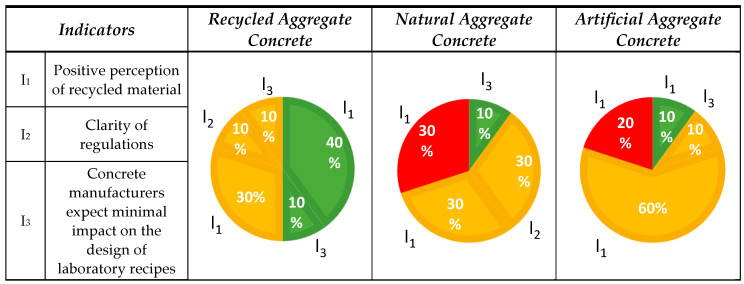
The classification of the published results presented in terms of the public perception aspect for the aggregates (RA, NA, and LA) and the concretes containing them. Publications assigned to indicators, RA: I_1_ [17,31,32,68,69,70,71]; I_2_ [72]; I_3_ [52], NA: I_1_ [31,42,73,74,75,76]; I_2_ [41,51,77]; I_3_ [61], LA: I_1_ [11,46,47,48,63,66,78,79,80]; I_2_ (none); I_3_ [44].

The assessment considered the societal impact by analyzing three interrelated indicators: social acceptance of material recycling (I_1_), regulatory transparency (I_2_), and minimal impact on the development of laboratory formulations (I_3_). The analysis of publications related to LA was complicated by the repetition of 6 out of 10 studies, which had previously been classified under environmental and economic aspects. For methodological consistency, no additional sources were searched. Based on the identified publications, no information on documentation transparency for this aggregate was recorded. The classification of the published results is presented in Figure 5.

Half of the analyzed publications (5 out of 10 (5/10)) were assessed as positive in terms of societal impact from RA [7,17,28,47,66]. The highest number of positive aspects, specifically four from all articles, was observed in the area of social readiness for changes necessary to protect the environment [17,31,32,68]. The analysis of NA’ societal perception showed a 40% worse result compared with the RA. Only 1 out of 10 opinions (1/10) was rated positively, relating to the minimal impact associated with modifications to laboratory formulations [61]. Similarly, for LA, only 1 out of 10 (1/10) publications directly addressed the positive perception of aggregates by society [11].

Indicator I_1_: Four publications on RA indicated that society values actions which reduce the amount of waste sent to landfills and limit the exploitation of natural resources [31,68]. Initiatives promoting and implementing eco-friendly solutions are supported [32]. Researchers strive to find optimal solutions for using various types of aggregates in concrete production. Advanced analysis methods such as life cycle assessment (LCA) and the VIKOR method are employed to integrate technical, economic, and environmental aspects [34]. Three publications highlighted solutions which support best practices in concrete recycling, promoting sustainable development and a circular economy [69]. One example is 3D printing using recycled concrete, which reduces labor and formwork costs, although further research is needed to mitigate cement’s environmental impact [62,70].

Three opinions on NA reported a negative societal perception. Ongoing consumption meets current societal needs but intensifies the extraction of aggregates, which is essential for the production of construction and infrastructure materials [31,73,74].

Two publications on LA identified a negative societal perception, as the processes for producing artificial aggregates, such as sintering at high temperatures (up to 1200 °C), result in significant energy consumption and greenhouse gas emissions [63,79]. One publication reported a positive perception for producing LA from industrial and agricultural waste, which reduces resource consumption, supports environmental protection, and binds CO_2_, positively impacting the societal perception and efforts to combat climate change [11]. Additionally, six publications addressed the potential positive impacts. The positive perception of LA production depends on the availability of appropriate raw materials meeting technological requirements, such as the combustible substance content, and on optimizing production processes using advanced research methods. Transparency in processes, public education, and adapting technology to local conditions and requirements are also critical, as they enhance acceptance and implementation effectiveness [46,47,48,66,78,80].

Indicator I_2_: Current knowledge suggests a potentially positive impact for recycled materials in construction, but it requires further exploration, such as through life cycle analyses. Further research will enable a better understanding, supporting informed decisions and broader use of these materials for sustainable development [72]. Three opinions on NA emphasized the potential positive impact of introducing transparent documentation. Various analyses are being developed to improve resource management, including the application of spatial scale theory. This approach facilitates precise policy modeling for NA management, accounting for local and regional differences in resource value, availability, and environmental and social costs [41,51,77].

Indicator I_3_: One publication on RA highlighted the potential positive impact on concrete technology development, stemming from the need to modify standard formulations to adapt them to the specific characteristics of recycled materials [52]. Another publication positively evaluated the impact of RA on mix designing, noting the potential for innovation, improved concrete properties, and support for sustainable construction [34]. One publication on NA suggested that the experience gained by conventional concrete manufacturers in using NA can effectively be applied to producing eco-friendly concrete with RA. This has been supported by numerous publications available in databases [61]. One publication on LA pointed out that laboratory formulations require a different approach compared with NA due to their specific properties, such as low density and high water absorption [44].

#### 3.1.4. Aspect No. 4: Quality Aggregate

**Figure 6:**
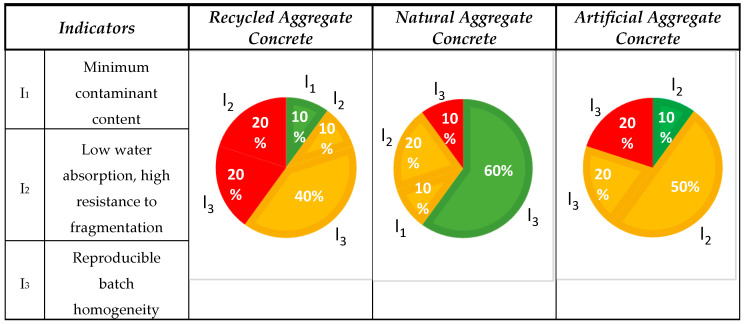
The classification of the published results, presented based on the quality aspect for RA, NA, and LA. Publications assigned to indicators, RA: I_1_ [81]; I_2_ [82,83,84]; I_3_ [85,86,87,88,89,90], NA: I_1_ [91]; I_2_ [92,93]; I_3_ [29,31,82,94,95,96,97], LA: I_1_ (none); I_2_ [7,11,46,50,63,98]; I_3_ [44,78,99,100].

The assessment took into account the impact of the parameters on quality by analyzing three interrelated indicators: the minimal amount of impurities (I_1_), reduced porosity (I_2_), and reproducible batch homogeneity (I_3_). The classification of the published results is presented in Figure 6.

Of all 10 analyzed publications for RA, only one indicated a positive result (1/10). In the case of NA, 6/10 publications were positive. LA received a positive feedback score of 1/10. The vast majority of the publications mentioned a possible positive impact. No publications were identified describing the impact of the degree of contamination on the concrete properties.

Indicator I_1_: The one publication on RA rated the minimum pollution rate. It was emphasized that material selection at the demolition stage reduces RA contamination and improves its quality. In the first stage, concrete was separated from impurities such as ceramics and wood, while in the second stage, old mortar and dust were removed, improving the aggregate’s density and water absorption [81].

Half of the analyzed publications mentioned the potential positive impact of aggregates on their quality, depending on several factors. Four publications pointed to the heterogeneity of aggregates, and one referred to their impact on porosity. Four publications presented negative opinions. NA also had one opinion indicating a potential positive impact, which depended on the level of contamination resulting from the crushing method and the origin of the rock [91].

Inicator I_2_: Regarding porosity, it was observed that RCA water absorption has a linear relationship with the porosity, grain density, and crushing resistance. An increase in water absorption and porosity leads to a decrease in the density of RCA and its resistance to mechanical damage [84]. Two negative publications emphasized the importance of grain structure on the degree of porosity, which is of crucial importance for RA quality. The amount of primary mortar surrounding the grain affects its properties, reducing the density and adhesion between the RA grain and the slurry as well as increasing water absorption, which creates a more porous structure. This porosity depends on the level of contamination and the crushing method used [82,83].

Two publications on NA indicated a potential positive impact. It is worth noting that the shape of the aggregate is largely dependent on the production process, including the type of crushing method used [92,93].

Five publications on LA indicated their potential positive impact on concrete production due to their low density, porosity, and lightness, making them an attractive material for lightweight constructions. The cold bonding method provides an effective recycling solution, enabling the production of aggregates with favorable strength and water absorption parameters [7,11,46,50,63].

Indicator I_3_: The heterogeneity of RA is a significant problem, as each demolition generates a different batch of material with varying properties. Nevertheless, it is possible to determine the properties of such aggregates, as evidenced by studies, such as by analyzing their compressive strength [101]. If the origin of RA is known, and its properties are studied, then its heterogeneity can be effectively managed [85,86,88]. Another two negative publications reported that an insufficiently controlled crushing process can lead to heterogenous aggregate, which in turn translates into difficulties in obtaining homogeneous concrete properties. This is particularly relevant in the case of concrete with higher quality requirements, where precise control of the material parameters is crucial for obtaining adequate mechanical properties and structural durability [87,89].

Six positive opinions on NAC mentioned that the properties of NA are so well known and sufficiently predictable that they can provide a solid basis for designing hybrid concrete mixes with the addition of RA, thus optimizing their mechanical properties and durability [29,82,94,97,102]. NA’ properties largely depend on their genesis, which in turn is related to a specific country and geological region [95]. The one negative opinion concerned the geometrical properties of aggregates, such as the size, shape, and inter-arrangement of grains, which are closely related to the production method, including the technological parameters of the crusher, the grinding process, and the properties of the material. The analysis showed that an appropriate selection of the fraction and the crushing process improved the crushing strength index (LA), which affects the quality of products such as concrete [96].

Two negative opinions regarding LA were related to the chemical composition of the substrates from which they were produced and the thermal conditions of the production process, which are crucial for the quality of this material. A low calorific value for the substrates reduces the process efficiency and requires modifications [44,50]. Two opinions indicated a potential positive impact, provided that local strategies are implemented to mitigate limitations in raw material availability, which could support sustainable concrete production [78,99].

#### 3.1.5. Aspect No. 5: Concrete Performance

**Figure 7:**
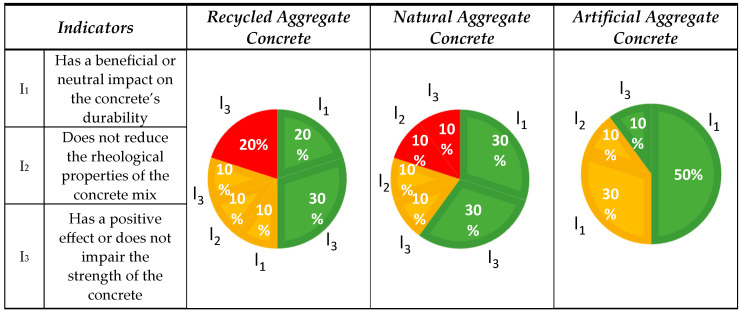
The classification of the published results presented for the aspect of concrete properties with the addition of aggregates (RA, NA, and LA). Publications assigned to indicators, RA: I_1_ [52,101,103]; I_2_ [104]; I_3_ [81,83,105,106,107,108], NA: I_1_ [59,61,109]; I_2_ [110,111]; I_3_ [101,110,112,113,114], LA: I_1_ [62,63,115,116,117,118,119,120]; I_2_ [121]; I_3_ [65].

The assessment took into account the impact of the quality of various aggregate types on the parameters of the concrete in which they were added by analyzing three interrelated indicators: a positive or neutral influence on the durability (I_1_), no significant impact on the rheology of the concrete mix (I_2_), and a positive or neutral influence on the compressive strength (I_3_). The classification of the published results is presented in Figure 7.

To summarize the scores for the RAC, five publications indicated a positive impact, three suggested a potential positive impact, and two mentioned a negative impact. Five out of 10 (5/10) publications indicated a positive result, which was four more than the opinion on aggregate quality. NA obtained positive feedback from 6 out of 10 publications. Two indicated a potential positive impact, and two indicated a negative impact. The concrete with LA received positive opinions from 6 out of 10 publications, while 4 indicating potential positive impacts.

Indicator I_1_: Two positive opinions indicated that RAC can achieve durability and mechanical properties comparable to NAC, provided the mix is properly designed [101,103]. A potentially positive effect on the durability of concrete can be achieved by using an appropriate degree of NA substitution, with the best results being obtained by using the processed structural concrete as a thick aggregate fraction [52].

Three positive aspects of using NA in concrete include the possibility of relying on many years of local experience, which makes it possible to adapt them to specific geological and climatic conditions [59,61,109].

Five positive opinions were found for LAC. Studies have shown that the mineral composition, as well as the structure and strength of aggregates have a significant impact on the mechanical properties and durability of high-quality concrete. The rough surface of LA facilitates a better bond with the cement slurry, which results in an increase in the concrete’s strength [120]. It may have a positive effect when an appropriate selection and degree of substitution of NA by LA makes it possible to improve the mechanical properties of lightweight concrete. Substitution of 20% improved the compressive strength of the concrete by 30%. LA from carbonized steel slag significantly affected the properties of the concrete, providing high compressive strength which increased with the curing time, reaching values from 36.2 MPa to 51.5 MPa [62,117]. The concrete achieved satisfactory values (20 MPa after 28 days) which are sufficient for non-load-bearing elements [63,118].

**Indicator** I_2_**:** One publication was attributed to an indicator related to the rheology of the concrete mix. RA has a potentially positive effect on the rheology of the mixture and depends on numerous factors. The properties of fresh concrete with RA depend on several variables. RA water absorption affects the workability, requiring water compensation or pre-hydration. The rate of water absorption determines the amount of additional water needed to obtain the right consistency. The RA fraction and its porosity increased the air level and reduced the density of the mixture. The density of RA and its percentage share in the mix’s affect the fresh density of concrete, which decreases along with an increasing share of RA. The air content increases when fine RA is applied, but it can be controlled with aerating admixtures. Water-reducing admixtures help improve workability, although their effectiveness depends on the properties of RA [104].

One negative opinion indicated that the presence of contaminants on the surface of NA particles, such as clay dust, leads to changes in the workability of the concrete mix [110]. One opinion indicated that using aggregate mixtures with varying particle shapes positively impacts the properties of the concrete mix. Aggregate with well-formed, rounded, and smooth particles improve concrete performance and reduce the water demand during mix preparation [111].

One of the key measures in designing laboratory mixes for concrete containing artificial lightweight aggregates is reducing the water-to-cement ratio (w/c) to mitigate their higher porosity [121].

Inicator I_3_: The highest number of positive results (three publications) for RAC was recorded for the indicator related to compressive strength. An appropriate technological regime, including limiting the replacement level of RCA, positively impacted the compressive strength of concrete. When up to 30% RCA was used, no significant deterioration in the mechanical properties was observed. However, above this level, a decline was noted. Reducing the water-to-cement ratio (w/c) in mixes with RCA further improved the compressive strength as well as the resistance to carbonation, chlorides, and sulfates [83,106,108]. Two publications on RAC showed the negative impact on the compressive strength results from the properties of the fine fraction of RA, such as high water absorption, particle agglomeration, and the presence of adjoining mortar. Owing to these features, concrete mixes with the addition of RCA tend to present lower compressive strength compared with concretes with fine NA [105,107]. The potentially positive impact of RA on the compression strength was described in one publication. Despite the overall negative impact of repeated concrete recycling on the aggregate quality and concrete durability, certain characteristics of concrete mixtures containing such aggregates have demonstrated improvement or stabilization after undergoing up to three recycling cycles. In other words, after several cycles, some concrete properties ceased to deteriorate and remained constant [81].

Three positive opinions for NA had a significant impact on the properties of concrete, providing predictable compressive strength and durability, which is a reference point for the assessment of alternative materials [101,112,114]. One opinion showed a potential positive effect, depending on the degree of rounding, which affects the quality of ITZ. Aggregate with a more rounded shape can improve interaction with the cement matrix, which translates into better mechanical properties, such as its compressive strength [113]. One positive opinion for LA from carbonated steel slag indicatesd a significant impact on the concrete properties, providing a high compressive strength which increased over time, reaching values from 36.2 MPa to 51.5 MPa [65].

### 3.2. The Results of a Multidimensional Assessment of the Desirable Characteristics of a Sustainable Product Based on the Authors’ Original Scoring System

The assessment was carried out according to the adopted scale of “ideal characteristics of aggregates” for the results obtained in relation to individual types of aggregates for five aspects. The summary referred to the answers to the questions posed in Stage II, namely what impact a given aggregate has on a selected aspect. The data are included in Table 4. According to the authors’ scoring method, the total scores for each type of aggregate and the concrete containing them were as follows. Recycled aggregate (RA) received 16 points, indicating its significant potential in terms of environmental and economic aspects, although areas requiring improvement were identified, particularly in terms of technical quality. Natural aggregate (NA) scored 13 points, confirming its high technical quality and positive impact on concrete properties. However, this score was lowered by its negative environmental impact and higher production costs. Artificial aggregate (LA) also received 14 points, reflecting its favorable environmental impact but simultaneously revealing limitations related to social perception and technical quality. The ideal score of 25 points represents the maximum achievable fulfillment of sustainability criteria, but none of the analyzed aggregates reached this value. The results highlight that despite their notable advantages, each of the studied aggregates requires further optimization to fully meet sustainability requirements, enhance social acceptance, and improve technical performance.

**Figure 8:**
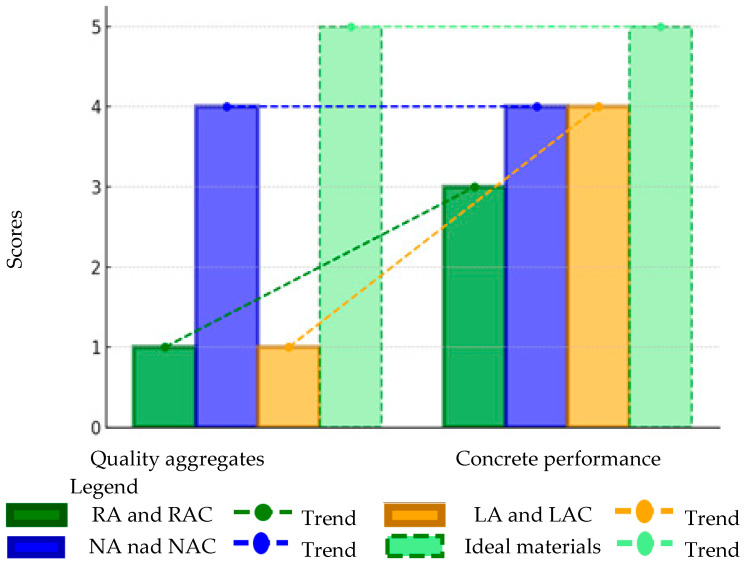
Assessment results for RA, NA, and LA and concretes containing them in relation to the desirable characteristics of a sustainable product, based on the authors’ scoring system for the aspects of aggregate quality and concrete properties.

When we considered the quality aspects of the aggregates and their impact on the quality of concrete, in both of these categories, the natural aggregates (NA) obtained a score of four, which positively demonstrates its high quality and the positive impact of this quality on the properties of concrete. Compared with NA, recycled aggregates (RA) performed much worse, obtaining the lowest rating of “1” (unacceptable). It is worth noting, however, that in the case of using RA in concrete, the assessment increased by two points, which indicates an improvement in the material parameters after its placement. A similar trend was also observed in the case of lightweight aggregates (LA). The assessment results are presented in Figure 8. The scoring scale represents different levels of meeting the desired characteristics of a sustainable product, while the trend lines for each type of aggregate illustrate the overall tendencies in the assessment results.

For the environmental aspect, all 10 opinions (100%) regarding concrete with recycled aggregates (RA) indicated their positive ecological impact. In the applied assessment scale, this result was classified as “5” (highly beneficial), confirming that RA meet the criteria of an ideal and sustainable material. Their application in concrete allows for a significant reduction in the exploitation of natural aggregates, contributing to the protection of natural resources. The same result was obtained by the LA, although with a lower satisfaction rate of 80%. NA received the lowest amount of positive points due to the high level of CO_2_ emissions and damage to the ecosystem. Therefore, it was classified as satisfactory. For subsequent aspects, a decrease in the positive impact of RAC, NAC, and LAC on the economy and public perception was observed.

In terms of cost comparison, the RA took first place, exerting the greatest impact on reduction, which was rated as “4” (beneficial). The LA were ranked second, receiving a grade of “3” (acceptable). The lowest rating was given to the NA, mainly due to their high transport costs, with a result of “2” (satisfactory).

In terms of public perception, for the third time, more points were garnered by the RA, while in the lower scale of RA grades, they fell to an acceptable level;, as NA received a score of “1” (unacceptable). One of the positive opinions primarily highlighted the experience of the concrete producer who designs laboratory formulations using these aggregates. LA also received the worst grade of “1” (i.e., unacceptable). According to the data collected, the reason for this could have been a limited range of applications, which negatively affected its perception.

RA, which initially received the lowest rating of “1” (unacceptable) due to their heterogeneity and potential impurities, significantly improved the quality of the final material when used in concrete (RAC), achieving a score of “3” (satisfactory). This trend indicates an enhancement in the technical parameters of the RA after incorporation into concrete, particularly at the optimal proportion of 25–30%, where the properties of the concrete remained uncompromised.

**Figure 9:**
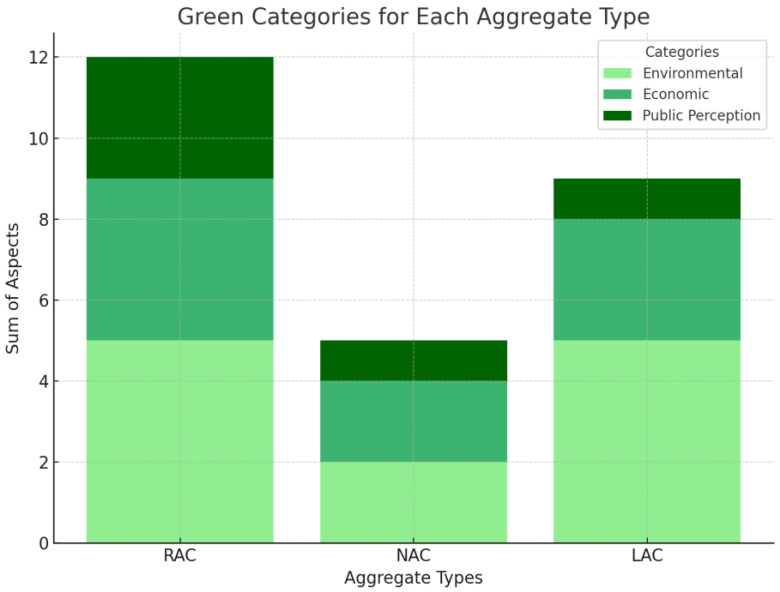
Assessment results for RA, NA, and LA and the concretes containing them in relation to the desirable characteristics of a sustainable product, based on the authors’ scoring system for the green aspects: environmental, economic, and public perception.

None of the aggregates met the ideal criteria in all aspects. RA stood out in environmental and economic terms, which suggests their growing potential, while NA remained the best choice in the context of the quality and properties of concrete. LA can be used in specific cases where low weight and insulation are important, but they require greater social acceptance. The point summary is presented in Figure 9.

The chart indicates that recycled aggregate concrete (RAC) was the most sustainable solution among the analyzed materials, particularly in terms of the environmental and economic aspects, highlighting its potential as a construction material which supports sustainable development. Lightweight aggregate concrete (LAC) also demonstrated significant potential but required further optimization in terms of costs to enhance its competitiveness. In contrast, natural aggregate concrete (NAC) achieved the lowest scores within the “green” categories, which could be attributed to its negative environmental impact and limited social acceptance, posing a challenge to its perception as a solution aligned with the principles of sustainable construction.

### 3.3. Identification and Analysis of the Key Sustainability Indicators Affecting the Maximum and Effective Use of Recycled Products

The comprehensive analysis conducted enabled the identification of key indicators influencing the sustainability of construction materials. The results were presented in the form of scoring and graphical visualization, allowing for evaluation of the advantages and disadvantages of each analyzed solution. This approach facilitated the identification of areas requiring improvement and the features most conducive to sustainable development.

When comparing the sustainability of RAC and NAC, two key issues can be distinguished. Firstly, for the overall score, RAC won with a score of 27 points, in comparison with 17 points scored by NAC. The detailed distribution of points is presented in Table 5. Secondly, we analyzed the points, which were divided into two groups of aspects; the first included the environmental, economic, and public perception aspects (green color shades on the graph), and the second included the quality of the aggregates and their impacts on the properties of concrete (gray color shades) with these materials. RAC played a key role in the first group, where it stood out as a sustainable construction product. The positive aspects in this group accounted for as much as 78% of its total assessment. In turn, NAC dominated in the second group due to its high quality, which positively affected the parameters of the concrete. For NAC, the first group of aspects covered only 29% of the total assessment, which indicates its limited impact in terms of environmental and economic sustainability.

The concrete with recycled aggregate achieved 54% of the ideal. This figure fell to 34% for the concrete with natural aggregate and 40% for the concrete with artificial aggregate. In terms of the environment, the recycled aggregate concrete scored the maximum number of points and was therefore ideal.

**Figure 10:**
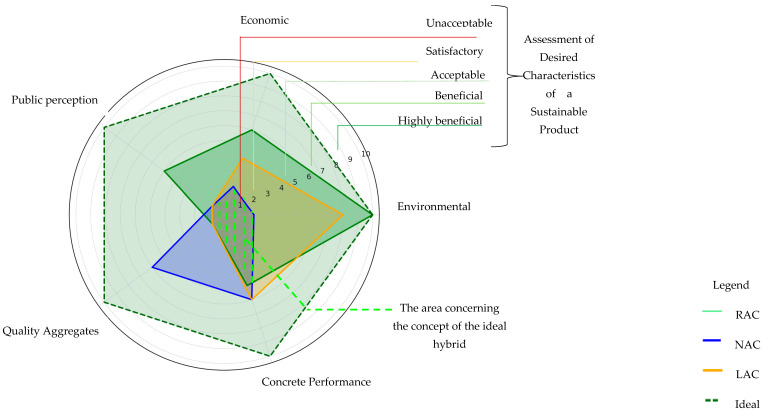
Visualization of the multidimensional analysis, highlighting a shared area among all aggregate types, which can be deemed critical for evaluating their sustainability.

The visualization of the multicriteria analysis included in Figure 10 identified a common area for all types of aggregates which can be considered crucial in assessing their sustainability. This area includes unacceptable characteristics common to all types of aggregates and concretes where they are added, indicating significant challenges which require solving. In the case of natural aggregates, the key problems are critical indicators related to environmental burden, such as the impact of extraction and CO_2_ emissions. For recycled aggregates and artificial aggregates, the lowest-quality parameters remain a challenge. From this perspective, the concept of hybrid concrete emerges as a potential model balancing these limitations. Recycled aggregate (concrete crushed rubble) obtained the highest score in the summary of all the analyses.

The division of the feasibility study into four stages was designed to ensure a clear and structured process, leading to the results of the multicriteria analysis. Expanding the analysis by including two additional aspects related to the quality of aggregates and the concretes containing them, combined with three key aspects of life cycle analysis—environmental, economic, and social—enabled a more comprehensive and holistic assessment of sustainability. The synergy between these aspects, visualized through multicriteria analysis, was crucial for identifying the optimal solution, which proved to be the concept of hybrid concrete, considered the closest option to the ideal of a sustainable product.

The application of an evaluation method incorporating the ideal characteristics of recycled aggregate concrete (RAC), natural aggregate concrete (NAC), and lightweight aggregate concrete (LAC) allowed for a precise definition of the expected features of the ideal product. This approach facilitated a detailed determination of the levels of individual parameters for each of the analyzed materials.

In conclusion, the authors’ multicriteria analysis was recognized as an appropriate and effective tool for achieving the objective outlined in the article. Its principles can serve as a foundation for individual analyses supporting decision making regarding the use of recycled aggregates (RA) as a substitute for natural aggregates (NA) to protect the natural environment.

## 4. Discussion

On the basis of Figure 8, Figure 9 and Figure 10, scientists identified some common areas in which recycled aggregate (RA) can be substituted for natural aggregate (NA) without significant deterioration of the concrete parameters while maintaining the benefit of sustainable development. On this basis, the important role of hybrid concrete as a solution combining the advantages of both types of aggregates was again emphasized. Other relevant aspects include the following:

1. Optimization of the amount of RA substitute in the partial exchange for NA

NA excel in technical stability, while RA and LA gain significance through chemical and mechanical processes occurring during the mixing and setting of concrete [81]. The practiced degree of substitutability bringing positive effects to the mechanical properties of RAC is 25–30% [29]. We observed a similar analogy in mineral-asphalt mixtures, emphasizing the importance of precise adjustment of RA proportions to achieve optimal parameters in both materials [6]. The optimal addition of RA effectively mitigates the issue of its natural heterogeneity.

2. RA quality assessment

RA quality assessment is crucial. The standard [25,26] allows the use of coarse aggregate in concrete, but the requirements for its quality remain liberal. In order to promote the potential of this aggregate in concrete more effectively, it would be useful to develop and supplement generally applicable technical documents with precise assessment criteria [12,34].

3. Life cycle cost analysis

The production of cement has the greatest impact on limiting the amount of CO_2_ equivalent. Portland cement accounts for 74–81% of the total CO_2_ emissions of concrete. In comparison, the reduction of CO_2_ emissions is 8%. Perhaps the attention of cement producers is focused on solving problems related to high emissions, whereby the potential of recycled aggregates is insufficiently perceived. LCA is, therefore, a tool which supports making conscious decisions in terms of choosing a sustainable solution [29].

4. Promotion of best practices

Instead of treating construction and demolition waste (CDW) as a problem, it should be seen as a secondary raw material with the potential to generate economic value (e.g., by recycling and producing new building materials). Such a change of approach supports the circular economy, reducing resource waste and the environmental impact [17]. Another example could be enhancing industrial symbiosis through the effective exchange of information, resources, and technologies between companies and organizations [122]. An example of the successful use of coarse recycled aggregate in Sardinia also highlights the importance of synergy between entities [123].

5. Additives supporting hybrid solutions

A supporting solution for the hybrid combination of NA and RA can involve advanced additives, such as nanomaterials, particularly nano-silica (NS), whose application has been shown to positively influence the durability, microstructure, and mechanical properties of RAC [124]. Other studies indicate that hybrid fibers can support the application of RAC in construction engineering, further enhancing its properties under dynamic loads [125,126]. Such additives can also come from recycling, such as fibers derived from car tires [127].

## 5. Conclusions

The multicriteria analysis, developed based on the feasibility study, enables a comprehensive assessment of the synergy between key aspects defining the sustainability of products. It serves as an advanced tool supporting the decision-making process, particularly in estimating realistic financial and energy savings. However, it should be emphasized that the effective use of this analysis requires an individualized approach tailored to specific conditions and requirements. A holistic approach, which considers all critical features in an integrated rather than isolated manner, allows for a more thorough and reliable evaluation of their suitability and potential impact:

1. Concrete with recycled aggregate, obtaining the highest score in the sustainability assessment as part of a comprehensive feasibility study, was considered the most advantageous solution among those analyzed, achieving the “green” distinction.
2. Currently, no product in the world has been identified which would meet all of the desired characteristics of a sustainable product, referred to in this article as “ideal characteristics of aggregates for concrete”. The concrete with recycled aggregates achieved 54% in relation to the ideal. This figure fell to 38% for the concrete with natural aggregates and 40% for the concrete with artificial aggregates.
3. The visualization of the results of the advanced analysis identified a common area for all types of aggregates which can be considered crucial in assessing their sustainability. This area includes unacceptable characteristics shared by all types of aggregates and the concretes they were added to, highlighting significant challenges which require resolution. From this perspective, the concept of hybrid concrete emerges as a potential model which could balance these limitations.

To explain why RA are not widely used on an industrial scale, we proposed a revision of the paradigm which considers natural aggregate (NA) concrete as the most sustainable solution. The analysis showed that despite its advantages, NA concrete significantly deviates from the ideal of a sustainable material, undermining its full compliance with the demands of modern sustainable development.

Further pilot studies will focus on validating the multicriteria analysis tool in real construction projects to assess its practical applicability and effectiveness in identifying sustainable solutions.

## Figures and Tables

**Table 1:** Glossary of subject-related abbreviations.

No.	Abbreviation	Explanation of the Abbreviation
1	RA	Recycled Aggregate
2	NA	Natural Aggregate
3	LA	Light Aggregate
4	RAC	Recycled Aggregate Concrete
5	NAC	Natural Aggregate Concrete
6	LAC	Lightweight Aggregate Concrete
7	CDW	Construction and Demolition Waste
8	CE	Circular Economy
9	LCA	Life Cycle Assessment

**Table 2:** Desirable characteristics of a sustainable construction product (key features).

Aspect	Indicator 1	Indicator 2	Indicator 3	Impact Description
Environmental	Reduces CO_2_ emissions	Reduces the use of natural resources	Reduces waste storage	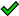 Reducing CO_2_ emissions, decreasing resource consumption, and minimizing waste storage support sustainable development
Economy	Reduces production costs	Reduces transportation costs	Reduces costs associated with waste storage	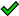 Reducing production, transportation, and waste storage costs improves profitability
Public perception	Positive perception of recycled materials	Clarity of regulations	Concrete manufacturers expect minimal impact on the design of laboratory recipes	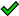 Positive perception of recycled materials, clear regulations, and minimal impact on formulations encourage the adoption of innovations
Quality aggregates	Minimum contaminant content	Low water absorption, high resistance to fragmentation	Reproducible batch homogeneity	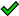 Low contaminant content, low water absorption, high resistance to fragmentation, and consistent batch homogeneity improve the quality of concrete
Concrete performance	Has a beneficial and neutral impact on the concrete’s durability	Does not reduce the rheological properties of the concrete mix	Has a positive effect or does not impair the strength of the concrete	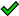 A positive impact or lack of deterioration in the durability, rheological properties, and strength of concrete increases the longevity and quality of constructions

**Table 3:** Assessment on the scale of ideal characteristics of aggregates for concrete: Stage III.

Percentage Range	Points	Interpretation of Points
80–100%	5	Highly beneficial
60–79%	4	Beneficial
40–59%	3	Acceptable
20–39%	2	Satisfactory
0–19%	1	Unacceptable

**Table 4:** Summary of points and scores based on “ideal characteristics of aggregates”.

Aspect	Recycled Aggregate (RA)	Natural Aggregate (NA)	Artificial Aggregate (LA)	Ideal
Environmental	5	2	5	5
	(highly beneficial)	(satisfactory)	(highly beneficial)	
Economic	4	2	3	5
	(beneficial)	(satisfactory)	(acceptable)	
Public prerception	3 (acceptable)	1 (unacceptable)	1 (unacceptable)	5
Quality aggregates	1	4	1	5
	(unacceptable)	(beneficial)	(unacceptable)	
Concrete performance	3 (acceptable)	4 (beneficial)	4 (beneficial)	5
Total points	16	13	14	25

**Table 5:** The distribution of classification points based on the conducted evaluation concerning the desired characteristics of a sustainable product.

Aspect	Recycled Aggregate (RA)	Natural Aggregate (NA)	Artificial Aggregate (LA)	Ideal
Environmental	10	2	8	10
Economic	6	2	4	10
Public prerception	5	1	1	10
Quality aggregates	1	6	1	10
Concrete prerformance	5	6	6	10
Total points	27	17	20	50

## Data Availability

No new data were created or analyzed in this study.
